# Conjugative Transposons and Their Cargo Genes Vary across Natural Populations of *Rickettsia buchneri* Infecting the Tick *Ixodes scapularis*

**DOI:** 10.1093/gbe/evy247

**Published:** 2018-11-06

**Authors:** Rachael Hagen, Victoria I Verhoeve, Joseph J Gillespie, Timothy P Driscoll

**Affiliations:** 1 Department of Biology, West Virginia University; 2 Department of Microbiology and Immunology, University of Maryland School of Medicine

**Keywords:** black-legged tick, integrative conjugative element, RAGE, REIS, droplet digital PCR, spotted fever group rickettsiae

## Abstract

*Rickettsia buchneri* (formerly *Rickettsia* endosymbiont of *Ixodes scapularis*, or REIS) is an obligate intracellular endoparasite of the black-legged tick, the primary vector of Lyme disease in North America. It is noteworthy among the rickettsiae for its relatively large genome (1.8 Mb) and extraordinary proliferation of mobile genetic elements (MGEs), which comprise nearly 35% of its genome. Previous analysis of the *R. buchneri* genome identified several integrative conjugative elements named Rickettsiales amplified genomic elements (RAGEs); the composition of these RAGEs suggests that continued genomic invasions by MGEs facilitated the proliferation of rickettsial genes related to an intracellular lifestyle. In this study, we compare the genomic diversity at RAGE loci among sequenced rickettsiae that infect three related *Ixodes* spp., including two strains of *R. buchneri* and *Rickettsia* endosymbiont of *Ixodes pacificus* strain Humboldt, as well as a closely related species *R. tamurae* infecting *Amblyomma testudinarium* ticks. We further develop a novel multiplex droplet digital PCR assay and use it to quantify copy number ratios of chromosomal *R. buchneri* RAGE-A and RAGE-B to the single-copy gene *gltA* within natural populations of *I. scapularis*. Our results reveal substantial diversity among *R. buchneri* at these loci, both within individual ticks as well as in the *I. scapularis* population at large, demonstrating that genomic rearrangement of MGEs is an active process in these intracellular bacteria.

## Introduction

The genus *Rickettsia* (*Alphaproteobacteria*: Rickettsiaceae) contains over thirty recognized species, ranging from serious human pathogens to species with no discernible pathogenicity (Beier-Sexton et al. 2015). All characterized rickettsiae are obligate intracellular bacteria that enter a host cell by induced phagocytosis, quickly lysing the phagocytic vacuole and residing primarily in the host cytosol (Winkler 1986); unlike many other cytosolic endoparasites, however, rickettsiae are entirely dependent on the host cell for replication and cannot replicate extracellularly (Ray et al. 2009). *Rickettsia* spp. exhibit a core metabolic capability that is surprisingly conserved but significantly reduced compared with other members of the Rickettsiales, requiring >50 predicted host nutrients and cofactors to survive (Driscoll et al. 2017).

*Rickettsia buchneri* (Kurtti et al. 2015; formerly *Rickettsia* endosymbiont of *Ixodes scapularis*, or REIS) is a transovarially transmitted endoparasite of the black-legged tick *Ixodes scapularis*, a common parasite of humans, companion animals, and wildlife throughout the Eastern United States and a medically important vector of various human pathogens including *Borrelia burgdorferi* (Lyme disease), *Anaplasma phagocytophilum* (anaplasmosis), and *Babesia microti* (human babesiosis). *Rickettsia buchneri* resides primarily in the ovarian tissues of adult female *I. scapularis* (Munderloh et al. 2005); it has no known pathogenicity in vertebrates and does not appear to undergo horizontal transmission to other eukaryotic hosts. The widespread distribution of rickettsiae in natural populations of *I. scapularis* has been well documented (Magnarelli et al. 1991; Noda et al. 1997; Benson et al. 2004); however, the ecological significance of these rickettsiae, particularly their potential impact on the fitness of their tick host, remains relatively unknown.

Previously, we described the extraordinary proliferation of mobile genetic elements (MGEs) in the accessory genome of *R. buchneri* infecting the Wikel colony of *I. scapularis* (Gillespie et al. 2012; hereafter, *R. buchneri* str. Wikel). One MGE of particular note is the Rickettsiales amplified genomic element (RAGE), first described in the genomes of *Orientia tsutsugamushi* str. Ikeda and Boryong, members of the scrub typhus group Rickettsiaceae (Cho et al. 2007; Nakayama et al. 2008). RAGEs belong to the integrative conjugative element (ICE) family of MGEs and serve as hotspots for multigene insertions (Blanc et al. 2007). RAGEs comprise a remarkable 9% of the *R. buchneri* str. Wikel chromosome, including seven complete or near-complete modules encoding F-like type IV secretion systems (F-T4SS); there are also two plasmid-encoded RAGEs that also encode F-T4SS but do not contain integrase genes.

RAGEs have been shown to harbor genes shared across diverse intracellular bacteria, including *spoT* stringent response regulators and *tlc* nucleotide transporters (Schmitz-Esser et al. 2004; Blanc et al. 2007; Gillespie et al. 2012), emphasizing their potential role in facilitating the spread of the bacterial mobilome—notably genes involved in adaptation to an intracellular lifestyle. Two RAGE loci (RAGE-A and RAGE-B) in *R. buchneri* str. Wikel are particularly interesting, as they contain multigene insertions with potentially significant functional implications. RAGE-A contains a large, 18-gene insertion between the F plasmid-like and Ti plasmid-like *traD* genes of its T4SS. This insert is dominated by genes typical of Gram-positive aminoglycoside antibiotic (AGAB) synthesis clusters (Shaw et al. 1993); it also contains a gene encoding a multidrug resistance transporter (MdlB) homologous to the *mldB* gene found in the temperate phage WO-B from various *Wolbachia* spp. RAGE-B harbors a smaller, 5-gene insertion that has replaced several canonical T4SS elements between its *traC* and *traAI* relaxase genes. The RAGE-B insert is primarily composed of hypothetical genes, with a single putative type I polyketide synthase (PKSI) not found in any other sequenced rickettsiae.

RAGEs are not confined to the *R. buchneri* genome, although it is the only known species with evidence of multiple intact RAGEs (Nakayama et al. 2008; Gillespie et al. 2015). Two other tick-borne rickettsiae (*R. bellii* strains RML369-C and OSU 85-389, and *R. massiliae* strain MTU5) each contain a single intact RAGE (RAGE-Br, RAGE-Bo, and RAGE-Ma, respectively), *R. peacockii* harbors a RAGE (RAGE-Pe) that appears to be partially degraded, and *R. felis* str. LSU-Lb contains a plasmid-encoded RAGE (RAGE-pLb). In addition, incomplete RAGE-like elements are found scattered across many rickettsial genomes. Strikingly, the RAGEs from *R. bellii* and *R. felis* are highly similar to *R. buchneri* RAGEs Be and p3, respectively, despite the distant relatedness between these rickettsiae (Gillespie et al. 2015). Furthermore, RAGE-pLb contains a gene that encodes a nonalphaproteobacterial DUF1016 protein also found duplicated in the *R. felis*, *R. bellii*, and *R. buchneri* chromosomes. Taken together, these data suggest that RAGEs are actively mobile and facilitators of gene exchange among diverse bacteria. The impact of RAGEs on the dissemination of potentially functional multigene cassettes, and particularly their amplification in the *R. buchneri* genome, remains to be explored.

In the current study, we focus on two primary questions. First, is the amplification of intact RAGEs associated with rickettsiae from *Ixodes* ticks in general or *I. scapularis* specifically? Second, what is the genomic diversity at RAGE loci among natural populations of *R. buchneri*? Accordingly, we use comparative genomics to investigate the distribution of RAGE loci across newly sequenced genomes of *Ixodes*-borne rickettsiae, including a natural isolate of *R. buchneri* (Kurtti et al. 2015; hereafter, *R. buchneri* str. ISO7). Additionally, we develop a novel dye-based, droplet digital PCR (ddPCR) multiplex assay to measure copy number ratios of two marker genes from the multigene insertions of *R. buchneri* RAGE-A and RAGE-B to the conserved, single-copy gene *gltA* in individual *I. scapularis* ticks. Our results indicate that the amplification of intact RAGEs appears to be specific to *R. buchneri*. We also describe several novel RAGEs including RAGE-G, a close relative of RAGE-A and RAGE-B that is present only in *R. buchneri* str. ISO7. Furthermore, we show that RAGE-B from *R. buchneri* str. ISO7 contains at least eight additional genes compared with *R. buchneri* str. Wikel; this expansion is dominated by genes typical of nonribosomal peptide synthesis (NRPS) and PKS clusters (Wang et al. 2014). Finally, our ddPCR assay uncovers surprising diversity in RAGE-A and RAGE-B copy number, both within individual *I. scapularis* ticks as well as across a broader natural population. Taken together, our results support a model of active lateral transfer and recombination at RAGE loci across *R. buchneri* strains that may have implications for the acquisition of novel functionality by this endoparasite.

## Expansion of Intact RAGEs is Restricted to *R. buchneri*

**Fig. 1.**
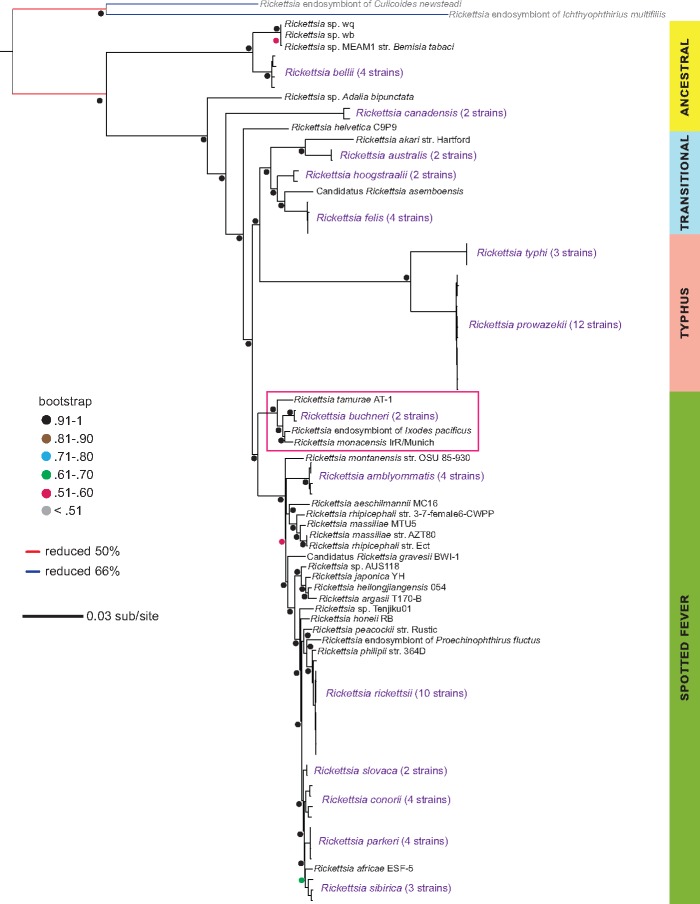
*—Ixodes-borne* *Rickettsia* spp. form a monophyletic clade within the traditional spotted fever group rickettsiae. Phylogenetic placement of the *Ixodes*-borne rickettsiae used in this study (red box) was inferred using maximum likelihood (*n* = 181 single copy orthologs; see text for details). Support values are based on 1,000 pseudoreplications. Strain-level variation is not described for monophyletic species; these branches are labeled in purple. Torix and Megaira group representatives (gray labels) have been shown to be basal to the *Rickettsia* (Schrallhammer et al. 2013; Pilgrim et al. 2017) and were used to root the tree. See supplementary table S1, Supplementary Material online, for relevant information identifying the genomes and loci used in this reconstruction.

Robust phylogeny estimation using 181 single-copy, core orthologs places the four sequenced Ixodes-borne rickettsiae—*R. monacensis* IrR/Munich (*I. ricinus*); *Rickettsia* endosymbiont of *Ixodes pacificus* strain Humboldt (REIP; *I. pacificus*); *R. buchneri* strain ISO7 (*I. scapularis*), and *R. buchneri* strain Wikel (*I. scapularis*)—within a monophyletic lineage subtended by *R. tamurae* strain AT-1 (Sentausa et al. 2014), a human pathogen (Imaoka et al. 2011) transmitted by the tick *Amblyomma testudinarium* (fig. 1). Based on these results, and the relatively unknown distribution of RAGEs across these closely related species, we focus our analyses described below on these five genomes.

**Fig. 2.**
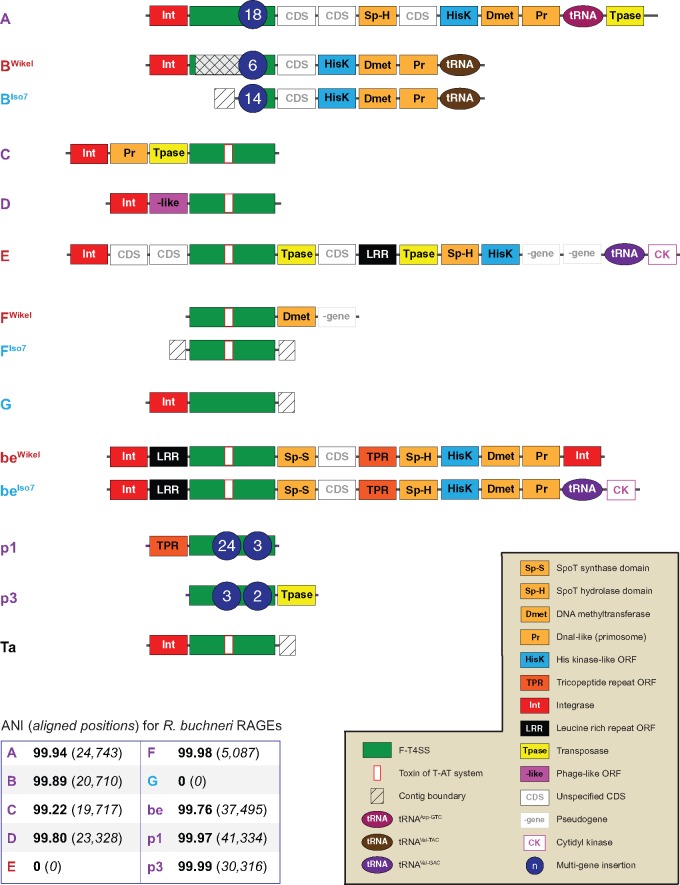
—Both sequenced isolates of *Rickettsia buchneri* contain multiple intact RAGE loci with evidence of genomic rearrangement. Gene order and functional annotations for each RAGE is shown in cartoon representation; see the included key (bottom right) for abbreviations and color scheme. RAGEs that are syntenic between isolates are labeled in purple. When two RAGEs differ between isolates, the *R. buchneri* str. Wikel variant is labeled in red and the *R. buchneri* str. ISO7 variant is labeled in blue. The novel RAGE-Ta described in this study is shown at the bottom and labeled in black. Gene order within the F-T4SS modules (green rectangles) and multigene insertions (blue ovals) are excluded for clarity; see supplementary tables S2 and S3, Supplementary Material online, for information on all the RAGE genes and their accession identifiers. Average nucleotide identities and the total aligned positions for shared RAGEs are shown in the grid at the bottom left; an ANI of 0% is listed for RAGEs that are unique to one isolate or the other.

*Rickettsia buchneri* str. ISO7 was originally isolated from the ovaries of a female *I. scapularis* removed from a dog in MN, United States (Kurtti et al. 2015). Its genome contains versions of at least six of the seven intact chromosomal RAGEs originally identified in *R. buchneri* str. Wikel, plus both plasmid-borne RAGEs (fig. 2 and supplementary table S2, Supplementary Material online). Furthermore, with a few notable exceptions (discussed below), there is a high degree of concordance between the two *R. buchneri* genomes in both average nucleotide identity (ANI) and gene order at their shared RAGE loci. These results demonstrate that the expansion of intact RAGEs in *R. buchneri* is not a byproduct of tick colony inbreeding, but is likely to have ecological implications across natural populations of *I. scapularis*.

**Fig. 3.**
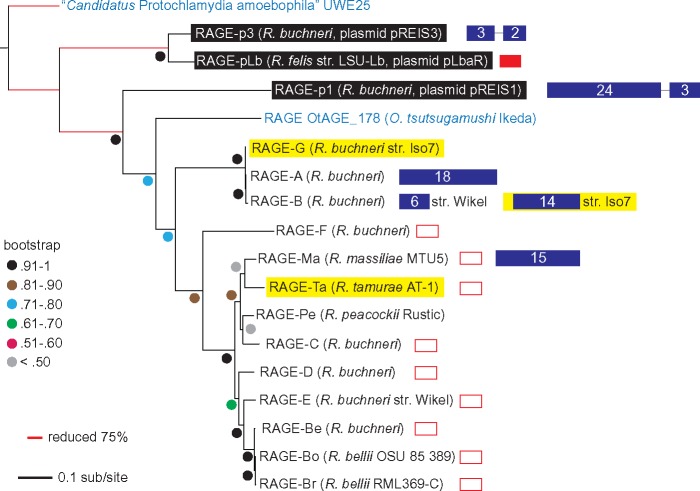
—RAGEs from disparate rickettsial lineages show close phylogenetic relationships. Phylogenetic placement of RAGEs newly described or characterized in the current study (highlighted in yellow) along with previously described intact RAGEs (Gillespie et al. 2015). The rickettsial lineage for each RAGE is shown in parentheses. Plasmid-borne RAGEs are highlighted in black, and multigene inserts indicated as blue rectangles. RAGEs that contain a toxin of T-AT system gene within their T4SS are denoted with an open red rectangle. The presence of an unknown (DUF1016) gene in place of the toxin in the RAGE-pLb T-4SS is indicated by a filled red rectangle. Phylogeny was inferred using maximum likelihood (see text for details) and support values are based on 1,000 pseudoreplications.

Our analysis also uncovered a heretofore undescribed intact RAGE (RAGE-Ta) within the genome of *R. tamurae* str. AT-1; consequently, *R. tamurae* is the fourth described rickettsial species (all tick-borne) with at least one intact RAGE (fig. 2 and supplementary table S3, Supplementary Material online). RAGE-Ta does not appear to contain a multigene insertion; however, the boundary of its parent contig (NZ_CCMG01000002.1) coincides with the end of its F-T4SS (*traD*) so an insertion cannot be definitively ruled out. Phylogenetic estimation suggests a shared ancestry for RAGE-Ta and RAGE-Ma of *R. massiliae*, an endoparasite of the tick *Rhipacephalus sanguineus* (fig. 3), providing yet another line of evidence supporting the mobility of RAGEs among disparate rickettsial lineages.

In contrast to *R. buchneri* str. ISO7 and *R. tamurae*, our analysis failed to uncover intact RAGEs in either *R. monacensis* or REIP genomes, despite a shared phylogeny with *R. buchneri* and a close phylogenetic relationship between their arthropod hosts. We consider it unlikely that RAGEs are masked by assembly gaps in these genomes, given the relatively high contiguity of their assemblies (*R. monacensis*: 1 contig; REIP: 7 contigs, *N*_50_ = 1.482 Mb). Both *R. monacensis* and REIP genomes do contain several putative *tra*-like and *int* loci, however, suggesting that genomic invasion by RAGEs has occurred in these species as has been described for other rickettsiae (Gillespie et al. 2012). Consequently, we conclude that the amplification of intact RAGEs appears to be limited to *R. buchneri* specifically. The significance of this amplification remains to be determined.

A detailed comparative genomics analysis of the sequenced *R. buchneri* genomes reveals evidence of recombination among different strains at several RAGE loci. In particular, RAGE-be in *R. buchneri* str. Wikel is flanked by *int* genes (EER21673.1, EER21704.1), but in *R. buchneri* str. ISO7 the phage-like *int* (EER21704.1) has been replaced by tRNA-Val^GAC^ and cytidyl kinase genes similar to the 5′ end of RAGE-E (99.74% ANI). Furthermore, we were unable to recover any other evidence of RAGE-E in *R. buchneri* str. ISO7, although our efforts here may have been hampered by the relative incompleteness of the ISO7 genome assembly (207 contigs, *N*_50_=13.5 kb). Intriguingly, we also uncovered evidence in the ISO7 strain of a novel RAGE (RAGE-G) not present in the original *R. buchneri* str. Wikel assembly (fig. 2). RAGE-G is closely related to RAGE-A and RAGE-B (fig. 3) except it does not appear to contain a multigene insertion, although the boundary of its parent contig (JFKF01000114.1) coincides with the end of its F-T4SS so any insertion may be lost in an assembly gap. The presence in *R. buchneri* of three distinct, closely related RAGEs (A, B, and G) with intact F-T4SS, strain-level variability, and taxonomically divergent multigene insertions also suggests that these MGEs are vehicles for the lateral transfer of genomic material from non-rickettsial organisms, as we discuss in more detail below.

## RAGE-B Contains a Hybrid NRPS/PKS Cluster

**Fig. 4.**
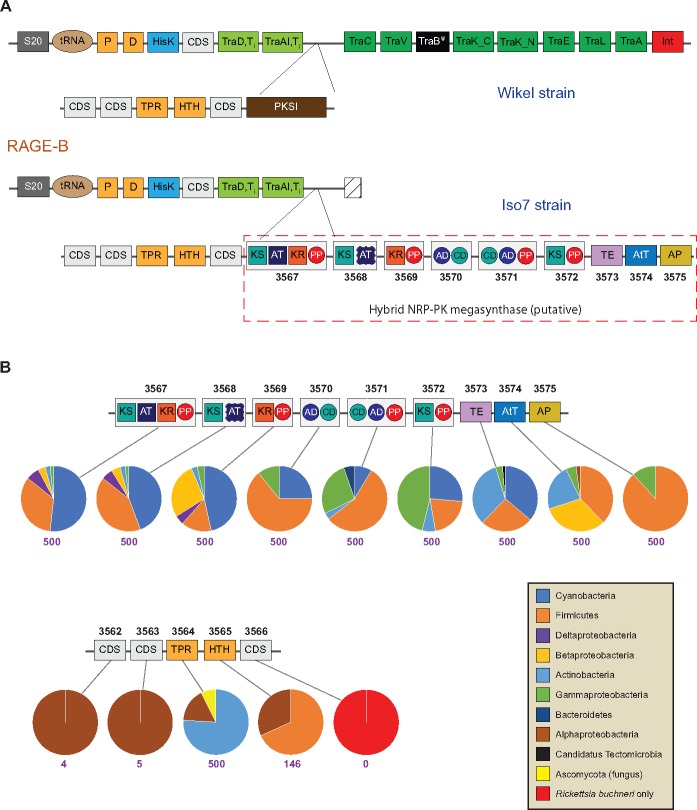
—RAGE-B harbors a multigene insertion that is distinctly nonalphaproteobacterial and strongly reminiscent of a hybrid NRPS/PKS cluster. (*A*) Comparison of gene order and functional annotation between RAGE-B variants from *Rickettsia buchneri* str. Wikel (top) and *R. buchneri* str. ISO7 (bottom). Functional symbols and color schemes generally follow figure 2. A red dotted line surrounds the nine genes that comprise the putative NRPS/PKS cluster in *R. buchneri* str. ISO7; the GenBank accession identifier for each protein is shown in black text below the corresponding cartoon representation (note: “KDO0” has been omitted from the start of each ID for brevity). Proteins in the putative cluster have been labeled with their predicted domains: KS, ketosynthase (teal); AT, acyltransferase (navy blue); KR, ketoreductase (orange); PP, phosphopantetheine binding (red oval); TE, thioesterase (lavender); AtT, acetyltransferase (blue); AP, aminopeptidase (gold). Proteins predicted to contain multiple domains are surrounded by a light gray rectangle. (*B*) Nonalphaproteobacterial origin for the 14 proteins of the RAGE-B multigene insertion. The putative NRPS/PKS cluster (top) and the remaining five genes of the RAGE-B insert (bottom) are shown in cartoon representation as in (*A*), along with pie charts showing the taxonomic distribution of their corresponding top BLASTP matches. Numbers below each pie chart (purple) indicate the total number of unique hits used to construct the distribution (maximum of 500).

RAGE-A and RAGE-B from *R. buchneri* str. Wikel both harbor multigene insertions with possible functional implications; accordingly, we further characterized these loci in the genome of the natural isolate *R. buchneri* str. ISO7. RAGE-A is essentially identical between the two *R. buchneri* genomes in both gene order and ANI (fig. 2); it harbors genes reminiscent of an AGAB cluster that has been discussed in detail previously (Gillespie et al. 2012). In contrast, the RAGE-B insertion from *R. buchneri* str. ISO7 contains at least eight additional genes (KDO03568.1–KDO03575.1) that were not evident in the *R. buchneri* str. Wikel genome (fig. 4). Unfortunately, the *R. buchneri* str. ISO7 insertion extends to the boundary of its parent contig (JFKF01000025.1); consequently we could not definitively identify the end of the insertion or recover the complete RAGE-B F-T4SS. We did identify several *tra*-like genes on isolated contigs, however, suggesting this region may not have been assembled completely in *R. buchneri* str. ISO7.

The eight novel RAGE-B genes from *R. buchneri* str. ISO7, together with the type I PKS (protein accession KDO03567.1) also found in the *R. buchneri* str. Wikel genome (protein accession EER22581.1), are strongly reminiscent of a hybrid NRPS/PKS cluster (fig. 4*A*). NRPS and PKS are large enzyme complexes responsible for the biosynthesis of diverse families of natural products with a wide range of biological activities, including antibiotics, immunosuppressants, toxins, and cytostatics (Felnagle et al. 2008). NRPS and PKS gene clusters are particularly common in bacteria and display extraordinarily diverse organization (Wang et al. 2014), often working in concert to produce highly specific secondary metabolites. They are also found less frequently in eukaryotes, and even more rarely in archaea; intriguingly, eukaryotic and archaean clusters appear to have arisen via horizontal transfer from bacteria (Gladyshev et al. 2008; Lawrence et al. 2011; Wang et al. 2014).

Sequence alignment and conserved domain analyses of KDO03567.1 (1,448 AA; 163 kDa) strongly suggest it is a canonical type I PKS (supplementary fig. S1, Supplementary Material online): it contains ketosynthase (KS), acyltransferase (AT), and ketoreductase (KR) domains, and both GHSxG (positions 753–758) and AFHS (positions 868–871) motifs crucial for cognate AT activity are completely conserved (Cheng et al. 2009). Furthermore, the C-terminus of KDO03567.1 contains a phosphopantetheine binding (PP) domain, a short post-translational modification site that is crucial for shuttling the growing peptide between domains of a NRPS/PKS complex (Robbins et al. 2016). The adjacent CDS (KDO03568.1, KDO03569.1) are composed of a KS domain (plus remnant AT residues) and a KR (plus PP) domain, respectively; together, these two proteins may constitute a second (AT-less) type I PKS (Cheng et al. 2009). The next two CDS (KDO03570.1, KDO03571.1) contain paired adenylation/condensation domains characteristic of NRPS elongation modules, while KDO03573.1 is a thioesterase (TE) that may function as a NRPS termination domain (Robbins et al. 2016). KDO03574.1 is an N-acetyltransferase (AtT) that may be a tailoring enzyme involved in modification of the final peptide; structural modification of NRP-PKs is commonplace, and AtTs in particular have been implicated in the NRPS-driven synthesis of penicillins and cephalosporins (Felnagle et al. 2008). Finally, KDO03575.1 (the last CDS on contig JFKF01000025.1) is an aminopeptidase (AP) with significant similarity to ClbP, a serine-reactive peptidase involved in the synthesis of the PK-NRP toxin colibactin in *E. coli* (Dubois et al. 2011).

Sequence similarity analyses (fig. 4*B*) support a decidedly non-alphaproteobacterial origin for genes in the NRPS/PKS-like cluster of RAGE-B, with predominant signals from Firmicutes, Cyanobacteria, and, to a lesser extent, *Gammaproteobacteria*. A similar pattern is seen in the tetracopeptide repeat (KDO03564.1) and helix-turn-helix (KDO03565.1) CDS of the RAGE-B insertion (the remaining three hypothetical CDS evince little or no significant similarity to any known sequences). It is intriguing to note that genes in the AGAB-like cluster of RAGE-A are similarly non-alphaproteobacterial in origin, with strong Firmicutes and Cyanobacteria signals (Gillespie et al. 2012). Furthermore, as described earlier, the T4SS modules of RAGE-A, RAGE-B, and the ISO7-specific RAGE-G are intact and very close phylogenetically (fig. 3). These observations raise the interesting possibility that RAGE-A/B/G may be a hotspot for the lateral acquisition of genes by *R. buchneri*, possibly as multiple invasions during repeated contact with soil environments (not unreasonable given the extended periods of time that ticks spend off-host). We are currently exploring in more detail the natural distribution of the genes being transferred by these RAGEs, and the composition of these RAGEs within broader populations of *R. buchneri*.

## RAGE Loci are Not Fixed in Intrahost *R. buchneri* Populations

**Fig. 5.**
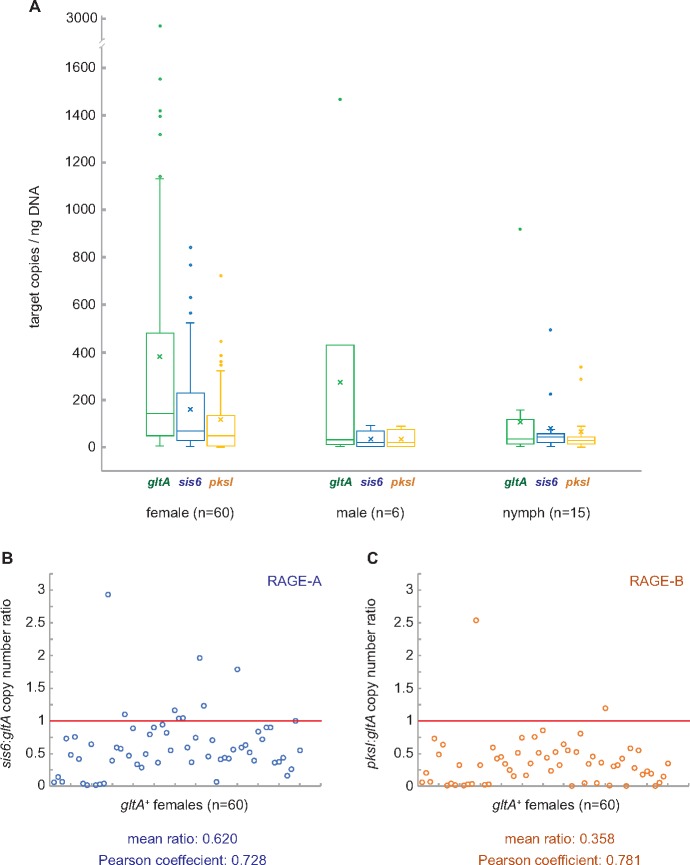
—RAGE-A and RAGE-B copy numbers are variable within individual *Ixodes scapularis* ticks. (*A*) Box-whisker plots of target copy numbers (normalized to DNA concentration) for *gltA* (chromosomal; green), *sis6* (RAGE-A; blue), and *pksI* (RAGE-B; orange) targets within female (*n*=60), male (*n*=6), and nymph (*n*=15) ticks. Each box displays the first and third quartiles (top and bottom of the box, respectively), the mean (*x*), and the median (horizontal line). Upper and lower whiskers indicate the local maximum and local minimum, respectively. Outliers are displayed as individual dots. (*B*) Copy number ratios of *sis6* (RAGE-A) to *gltA* (chromosomal) for *Rickettsia buchneri* populations within individual female *I. scapularis*. Each open circle (blue) represents the *sis6:gltA* ratio within a single tick. The expected ratio of 1 is shown by the red horizontal line. The mean ratio over all ticks and the Pearson coefficient of correlation between *sis6* and *gltA* copy numbers are shown below the chart. (*C*) Copy number ratios of *pksI* (RAGE-B) to *gltA* (chromosomal) for *R. buchneri* populations within individual female *I. scapularis*. Each open circle (orange) represents the *pksI:gltA* ratio within a single tick. The expected ratio of 1 is shown by the red horizontal line. The mean ratio over all ticks and the Pearson coefficient of correlation between *pksI* and *gltA* copy numbers are shown below the chart.

The divergence between sequenced genomes of *R. buchneri* strains Wikel and ISO7 described earlier suggested the possibility of ongoing natural variability at RAGE loci in this species. To investigate this possibility, we developed a ddPCR assay to quantify the distribution of RAGE-A and RAGE-B targets (compared with a *Rickettsia*-specific *gltA* target) among natural populations of *I. scapularis* ticks. We detected *Rickettsia gltA* in 94% of adult female *I. scapularis* sampled (74 out of 78), but only 76% of males and 49% of nymphs (table 1). Furthermore, mean *Rickettsia* abundance as determined by *gltA* copy number per nanogram of DNA was greater in infected female ticks compared with infected males (2.2×) and nymphs (3.5×), though these differences were not statistically significant likely due to high variability between individuals (fig. 5*A* and supplementary table S4, Supplementary Material online). The higher prevalence of *R. buchneri* in adult female *I. scapularis* that we report here likely reflects the expansion of *R. buchneri* populations in ovarian tissues as preparation for transovarial transmission. To our knowledge, this is the first direct quantification of *R. buchneri* populations from individual ticks across different life stages.

**Table 1:** Abundance and Distribution of Rickettsial Gene Targets across Collected *Ixodes scapularis* Ticks

Stage	Count of Ticks Collected^a^	% (count) of *gltA*^+^ Ticks	Mean Copies of *gltA* Per ng DNA^b^	Count of Ticks Tested for RAGEs A and B	% (count) of *gltA^+^ sis6*^−^*pks1^+^* Ticks	% (count) of *gltA^+^ sis6^+^ pks1*^−^ Ticks	% (count) of *gltA^+^ sis6*^−^*pks1*^−^ Ticks
Female	78	95% (74)	366.2*	60	5% (3)	25% (15)	5% (3)
Male	17	76% (13)	166.9*	6	0% (0)	0% (0)	0% (0)
Nymph	35	49% (17)	103.6*	15	20% (3)	13% (2)	7% (1)

Note.—The percentage of *gltA*^+^ ticks (column 3) and mean copies of *gltA* per ng DNA (column 4) were calculated from the total number of collected ticks (column 2). All other percentages were calculated from the number of ticks tested for RAGEs A and B (column 5).

a Samples without measurable DNA are not included in these counts.

b Samples with *gltA* copy number <10 were considered *gltA*^−^.

* Denotes a lack of significance (*p* < 0.05) between labeled groups, according to analysis of variance (see main text for details).

Next, we developed a multiplex ddPCR assay to simultaneously quantify copies of the *sis6* (RAGE-A; KDO03401.1) and *pksI* (RAGE-B; KDO03567.1) genes in individual *gltA*^+^ female *I. scapularis* ticks (*n* = 60). *sis6* and *pksI* were chosen because they are present in both sequenced strains of *R. buchneri*, they are predicted to be single copy genes, and they appear to be absent from all other *Rickettsia* spp. (and even more broadly, all *Alphaproteobacteria*). Our ddPCR results reveal considerable variability in *sis6*:*gltA* and *pksI*:*gltA* copy number ratios within individual ticks (fig. 5*B* and *C*; supplementary table S5, Supplementary Material online), suggesting a certain heterogeneity at these RAGE loci within populations of *R. buchneri* that infect individual ticks. Furthermore, the overall *pksI*:*gltA* copy number ratio (0.358) was just over half the *sis6*:*gltA* ratio (0.620), and both ratios were well below what we would predict for single copy genes (i.e., 1.0). Based on these results, we postulate that these RAGE loci are not fixed in *R. buchneri* populations; indeed, the intrahost diversity supports a degree of population selection acting at the level of the individual host, although it does not rule out host population effects. Intriguingly, RAGE-B appears to be less prevalent in *R. buchneri* populations compared with RAGE-A: in addition to a lower *pksI:gltA* copy number ratio, five times as many *gltA*^+^ ticks had undetectable levels of *pksI* compared with ticks with undetectable levels of *sis6* (table 1). The type of selection acting upon these loci, and whether it is the same for both RAGE-A and RAGE-B, remains to be determined.

It will be interesting to explore in more detail the dynamics of gene loss and movement in these RAGEs, particularly in light of their potential role in the synthesis of novel secondary metabolites. Clearly such compounds would be advantageous in the complex ecology of a soil microbiome (Tyc et al. 2017). It is possible that these multigene insertions are merely remnants of previously active synthesis clusters that are now undergoing gene loss and degradation in *R. buchneri*; nonetheless, they provide valuable insight into the mechanisms that affect the genetic makeup of vector-borne microbes. In any case, the results of our study lend further credence to a model wherein RAGEs represent an active mechanism for the horizontal acquisition of novel functionality by *R. buchneri* despite its cloistered lifestyle.

## Materials and Methods

### Genus-Level Phylogeny Estimation

Protein sequences (*n* = 113,470) for 87 sequenced rickettsial genomes (supplementary table S1, Supplementary Material online) were either downloaded directly from NCBI (*n* = 71), or retrieved as genome sequences from the NCBI Assembly database (*n* = 14) or via personal communication (*n* = 2; Lucy Weinert, University of Cambridge). For genomes lacking functional annotations (*n* = 16), protein models were predicted using the Rapid Annotation of Subsystems Technology (RAST) v2.0 server (Aziz et al. 2008). Ortholog groups (*n* = 3,278) were subsequently constructed using FastOrtho, an in-house version of OrthoMCL (Li et al. 2003), using an expect threshold of 0.001, percent identity threshold of 30%, and percent match length threshold of 50% for ortholog inclusion. A subset of single-copy families (*n* = 181) conserved across all 87 genomes were independently aligned with MUSCLE v3.8.31 (Edgar 2004) using default parameters, and regions of poor alignment were masked with trimal v1.4.rev15 (Capella-Gutiérrez et al. 2009) using the -automated1 option. All modified alignments were concatenated into a single data set (49,030 positions) for phylogeny estimation using RAxML v8.2.4 (Stamatakis 2006), under the gamma model of rate heterogeneity and estimation of the proportion of invariant sites. Branch support was assessed with 1,000 pseudoreplications.

### Identification of Novel RAGEs by Comparative Genomics

Ortholog groups constructed as part of the genus-level phylogeny estimation were initially screened for key RAGE components using the *R. buchneri* str. Wikel annotations, including: RAGE-associated integrase (7), toxin of T-AT system (5), TraE (9), TraW (6), TraU (6), and TrbC (6) proteins. Putative orthologs to these elements in the target genomes (*R. buchneri* str. ISO7, *Rickettsia* endosymbiont of *Ixodes pacificus*, *Rickettsia monacensis* IrR/Munich, and *R. tamurae*) were identified and the surrounding genomic regions inspected manually for additional RAGE-like components. To search for previously undescribed RAGE loci, the same RAGE-associated sequences from *R. buchneri* str. Wikel described earlier were used to query the four target genomes with BLASTP (Altschul et al. 1990), using default parameters. Putative matches were located in their respective genomes and the surrounding regions inspected manually for evidence of additional RAGE elements. Plasmid-borne RAGEs in *R. buchneri* str. ISO7 were identified and assembled manually. Finally, proteins annotated as transposase in the four target genomes were subjected to a similar manual inspection for the presence of additional RAGE elements. Average nucleotide identities (ANI) for the RAGEs shared between *R. buchneri* str. Wikel and str. ISO7 were calculated using megaBLAST (Goris et al. 2007).

To assess the origin of the *R. buchneri* str. ISO7 RAGE-B multigene insertion, protein sequences for the nine putative NRPS/PKS genes (KDO03567.1–KDO03575.1) plus the five additional genes of the insertion (KDO03562.1–KDO03576.1) were used as queries (BLASTP) against the nr database of NCBI (excluding models and environmental sample sequences). Default parameters for BLASTP were used except the scoring matrix was changed to BLOSUM45 and the expect threshold was set to 0.01. Taxonomic assignments for the top 500 hits to each protein were compiled into counts of unique genera with at least three hits, and summarized by taxonomic group.

To assemble the putative NRPS/PKS encoded in *R. buchneri* str. ISO7 RAGE-B, protein sequences for the nine genes (KDO03567.1–KDO03575.1) of the cluster were submitted as queries against the conserved domain database (CDD) of NCBI (Marchler-Bauer et al. 2017), using default parameters. To assess the conservation of critical residues in the AT domain of PKSI, the protein sequence from *R. buchneri* str. ISO7 (KDO03567.1) was used to query (BLASTP) the nr database of NCBI (default parameters). The top 40 matches (plus the two *R. buchneri* sequences) were aligned with MUSCLE v3.8.31 (default parameters) and used to construct a sequence logo (Crooks et al. 2004) encompassing the AT domain (alignment positions 400–900).

### RAGE Phylogeny Estimation

Protein sequences (*n* = 213) for 14 F-T4SS elements conserved across 18 complete or nearly complete RAGEs were independently aligned with MUSCLE v3.8.31 using default parameters, and regions of poor alignment were masked with trimal v1.4.rev15 using the -automated1 option. All modified alignments were concatenated into a single data set (5,320 positions) for phylogeny estimation using RAxML v8.2.4, under the gamma model of rate heterogeneity and estimation of the proportion of invariant sites. Branch support was assessed with 1,000 pseudoreplications. The 14 F-T4SS proteins used in the alignment were as follows: TraL, TraE, TraK, TraB, TraV, TraC, TraW, TraU, TrbC, TraN, TraF, TraH, TraG, and TraD_F.

### Tick Collections

*Ixodes scapularis* ticks (*n* = 130) were collected between 2014 and 2016 by direct submission from veterinary clinics in West Virginia (WV), United States, as part of a statewide surveillance program coordinated by the WV State Department of Epidemiology (Zoonotic Disease Division). Ticks were identified to species and life stage by morphological characteristics, then stored in 70% ethanol at −20 °C until DNA extraction, which was carried out within 3 months of collection.

### DNA Extraction

*Ixodes scapularis* adults and nymphs were immersed for 2–3 min in 10% bleach, 70% ethanol, and sterile H_2_O in succession to reduce surface contamination, then separated into individual tubes, frozen in liquid nitrogen, and homogenized with sterile plastic micropestles to pulverize exoskeleton and tissues. Total genomic DNA was subsequently extracted using the GeneJET DNA Extraction Kit (ThermoFisher) following the manufacturer’s suggested protocol for Gram-negative bacteria and eluted in 50 µl of sterile H_2_O. Final DNA concentrations were assayed by fluorimetric methods (Qubit 3.0; ThermoFisher) and DNA stored at −20 °C until ddPCR. Individual *I. scapularis* with measurable DNA (*n* = 112) were used to quantify *R. buchneri* and determine target gene ratios as described below.

### Quantification of *R. buchneri* in Individual Ticks

Copy numbers of a highly conserved, single-copy *R. buchneri* gene from individual *I. scapularis* DNA extractions (*n* = 112) were quantified using a QX200 ddPCR system (Bio-Rad Laboratories) following the manufacturer’s protocols for dye-based measurements. ddPCR enables absolute quantification of nucleic acids with greater precision and much higher tolerance of differences in amplification efficiencies between primer sets (Hindson et al. 2011). In ddPCR, a standard PCR reaction is prepared and subsequently partitioned into nanoliter droplets before running end-point PCR. The fluorescence of each droplet in the population is measured and binned into positive and negative droplets. The number of target DNA copies in the reaction is calculated from the fraction of positive droplets using Poisson statistics (Hindson et al. 2011).

In order to quantify *R. buchneri* in individual ticks, a 74-bp target region of the rickettsial citrate synthase (*gltA*) gene (Stenos et al. 2005) was amplified and visualized with EvaGreen, a nucleic acid stain similar to SYBR green but optimized for use with the QX200. The restriction enzyme BsaI (New England Biolabs) was added (2U/reaction) as per manufacturer specifications; restriction of genomic DNA is recommended in ddPCR in order to minimize potential inhibition of the polymerase due to increased viscosity in nanoliter droplets. NEBcutter (Vincze et al. 2003) was used to verify that BsaI does not digest the *gltA* amplicon. *Rickettsia amblyommatis* DNA (kindly provided by Kevin Macaluso, Louisiana State University) was used as a positive control. Each reaction contained 1 µl of DNA sample as template; since neither the *sis6* nor *pksI* target were sensitive to DNA concentration (supplementary table S6, Supplementary Material online), we did not normalize template to DNA concentration. The number of target copies in the reaction was subsequently normalized to nanograms of DNA to account for between-sample variability in tissue homogenization and extraction efficiency. Samples with less than ten copies of target per reaction were considered negative for *R. buchneri* as per the manufacturer’s recommendation; this conservative threshold excluded a small number of samples (*n* = 14) and was used to account for the low level of false positive droplets that may occur as a result of non-specific binding by the Evagreen dye. Primer sequences used in all ddPCR analyses can be found in supplementary table S7, Supplementary Material online.

### Impact of Tick Life Stage on *R. buchneri* Abundance

Individual *gltA*^+^*I. scapularis* with measurable DNA (*n* = 96) were further assessed for a potential relationship between tick life stage (female, male, nymph) and *R. buchneri* abundance. All statistical analyses were carried out in R (R Core Team 2017). *gltA* copy numbers were normalized to DNA concentration for each individual tick, and a one-way analysis of variance (ANOVA) with life stage as the factor was calculated (aov, TukeyHSD). Homogeneity of variances between treatments was confirmed by plotting residuals against the fitted values.

### Multiplex ddPCR Assay for *R. buchneri* Specific Targets

Individual *gltA*^+^*I. scapularis* females (*n* = 61) were simultaneously assessed for *sis6* (KDO03401.1; RAGE-A) and *pksI* (KDO03567.1; RAGE-B) using a multiplex ddPCR assay based on differential amplicon length. Primer sets for each target were designed using primer-BLAST (Ye et al. 2012) and cross-checked for possible off-target priming using BLASTN against the nr database of NCBI. Annealing temperatures for all primers were required to be within 1 °C of 60 °C to enable efficient multiplexing. Final amplicon lengths (*sis6*: 77 bp; *pksI*: 134 bp) were selected to maximize the size difference between the two targets, and the assay was optimized using a DNA sample that was strongly positive for both *sis6* and *pksI* targets. When visualized with EvaGreen, the two target amplicons yielded distinct subpopulations of droplets within the same reaction. Copy numbers for each target were quantified separately using QuantaSoft analysis software v1.7 (Bio-Rad Laboratories). As with *gltA* ddPCR described earlier, BsaI was added to each reaction mixture (2U/reaction) after verification with NEBcutter, to reduce possible effects of viscosity. 

## Supplementary Material

Supplementary data are available at *Genome Biology and Evolution* online.

## Supplementary Material

Supplementary Data
