# Supplementary material for: Conjugative Transposons and Their Cargo Genes Vary across Natural Populations of Rickettsia buchneri Infecting the Tick Ixodes scapularis
Source: Genome Biol Evol. 2018 Nov 6;10(12):3218–29. doi: 10.1093/gbe/evy247 (PMC6300072; doi:10.1093/gbe/evy247)
Supplement: Supplementary Data [file evy247_supp.zip › ALL_SUPPLEMENTARY_FIGURES.pdf]

Supplementary Figure S1. Conserved domain analysis of the PKSI protein of RAGE-B.

(**A**) Primary domains identified by CD-Search within the putative type I PKS in *R. buchneri* str. Iso7 (KDO03567.1). Each predicted domain is shown in context of the complete protein and labeled with the functional annotation, domain tag, and CDD ID (blue). The color scheme generally follows **Figure 4**. The solid yellow rectangle indicates the region analyzed in more detail in (B).

(B) Conservation of the acyltransferase motifs in KDO03567.1. Sequence logo calculated from the alignment of KDO03567.1 with 41 additional PKSI proteins identified by BLASTP. Only positions 700-900 of the alignment are shown, which corresponds to positions 574-739 in the *R. buchneri* str. Iso7 ortholog. The motifs critical for acyltransferase catalytic activity (Cheng 2009 REF) are indicated by solid blue bars.

Figure S1

A

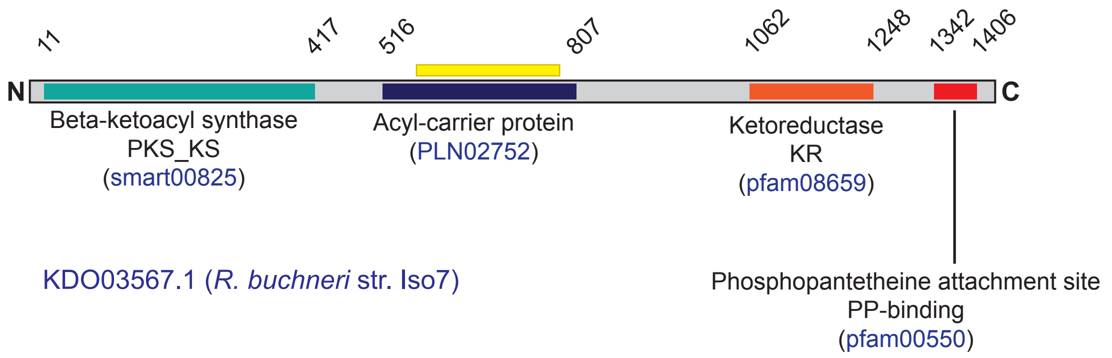
11
417
516
807
1062
1248
1342
1406
N
C
Beta-ketoacyl synthase
PKS_KS
(smart00825)
Acyl-carrier protein
(PLN02752)
Ketoreductase
KR
(pfam08659)
Phosphopantetheine attachment site
PP-binding
(pfam00550)
KDO03567.1 (R. buchneri str. Iso7)

B

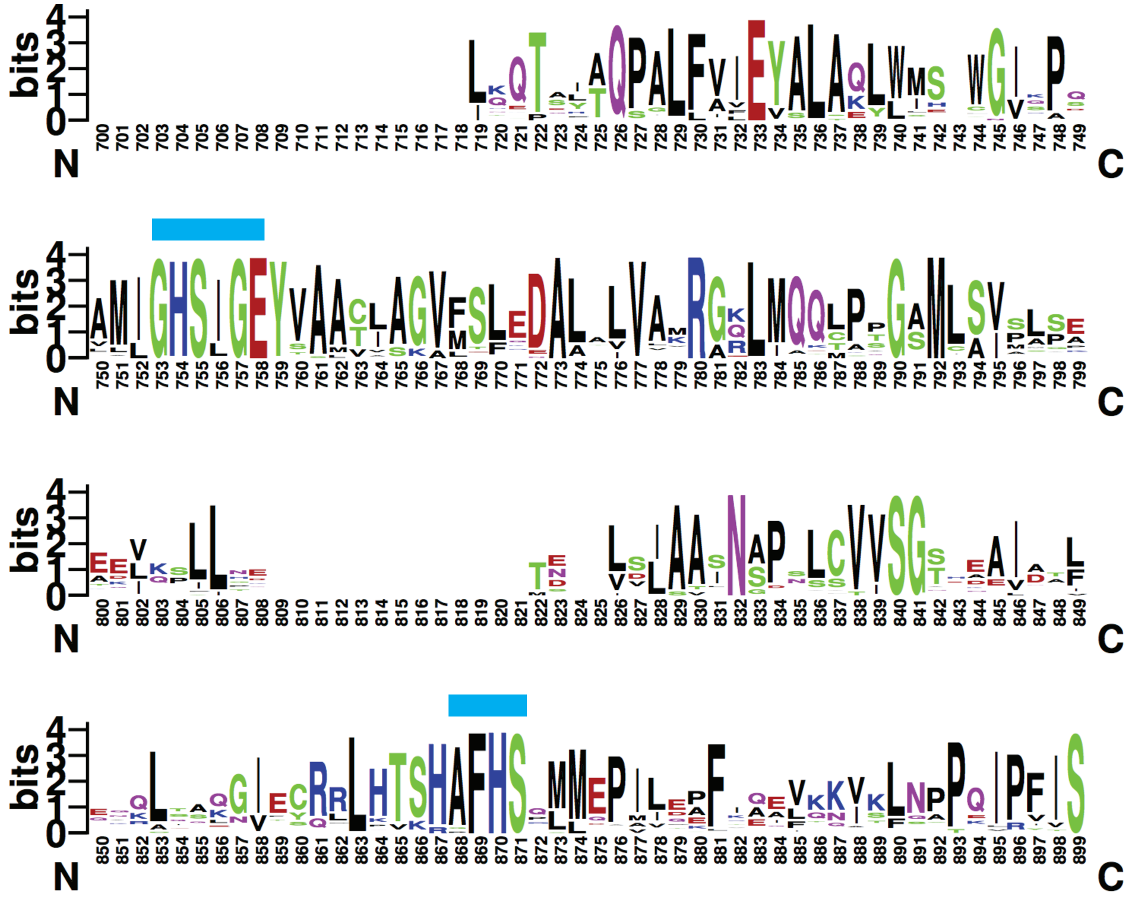
bits
N
C
