# Supplementary material for: Conjugative Transposons and Their Cargo Genes Vary across Natural Populations of Rickettsia buchneri Infecting the Tick Ixodes scapularis
Source: Genome Biol Evol. 2018 Nov 6;10(12):3218–29. doi: 10.1093/gbe/evy247 (PMC6300072; doi:10.1093/gbe/evy247)
Supplement: Supplementary Data [file evy247_supp.zip › Legends supplementary materials.docx]

**Additional information**

Supplementary information accompanies this paper:

**SUPPLEMENTARY FIGURE S1**. Conserved domain analysis of the PKSI protein of RAGE-B.

**SUPPLEMENTARY TABLE S1**. Accession identifiers for rickettsial genomes and orthologs used in this study.

**SUPPLEMENTARY TABLE S2**. Accession identifiers and genome arrangement of *R. buchneri* RAGEs.

**SUPPLEMENTARY TABLE S3**. Accession identifiers and genome arrangement of *R. tamurae* RAGE-Ta.

**SUPPLEMENTARY TABLE S4**. Statistical analysis of the relationship between tick life stage and *R. buchneri* abundance.

**SUPPLEMENTARY TABLE S5**. Raw target copy data for individual *I. scapularis* ticks.

**SUPPLEMENTARY TABLE S6**. Copy number of ddPCR targets with increasing input DNA concentration.

**SUPPLEMENTARY TABLE S7**. Primers used in ddPCR assays.
